# Circulating extra-cellular RNAs and atrial fibrillation: data from the TRACE-CORE cohort

**DOI:** 10.3389/fcvm.2025.1623112

**Published:** 2025-07-15

**Authors:** Katherine Tak, Darleen Lessard, Catarina I. Kiefe, Jane E. Freedman, Matthew Parker, Gerard P. Aurigemma, Kevin Donahue, David D. McManus, Khanh-Van Tran

**Affiliations:** ^1^ Department of Medicine, University of Massachusetts Medical School, Worcester, MA, United States; ^2^ Department of Quantitative Health Sciences, University of Massachusetts Medical School, Worcester, MA, United States; ^3^ Department of Medicine, Vanderbilt University Medical Center, Nashville, TN, United States

**Keywords:** atrial fibrillation, echophenotypes, microRNA, biomarker, adverse cardiac remodeling

## Abstract

**Background:**

Atrial fibrillation (AF) is the most common sustained arrhythmia and is linked to increased risk of stroke, heart failure, and mortality. Circulating extracellular RNAs (exRNAs), which regulate gene expression and reflect underlying biological processes, are potential biomarkers for atrial fibrillation.

**Methods:**

As part of an ongoing, larger study into extracellular RNAs (exRNAs) as potential biomarkers for cardiovascular disease, we analyzed exRNA profiles in a subset of 296 survivors of acute coronary syndrome (ACS) enrolled in the Transitions, Risks, and Actions in Coronary Events Center for Outcomes Research and Education (TRACE-CORE) cohort. A total of 318 exRNAs were quantified, selected *a priori* based on prior findings from the Framingham Heart Study. We assessed associations between circulating exRNAs and echocardiographic intermediate phenotypes relevant to atrial fibrillation (AF), including left atrial dimension, left ventricular (LV) mass, LV end-diastolic volume, and global longitudinal strain. Subsequently, we used logistic regression models to evaluate whether the exRNAs associated with these phenotypes were also associated with a history of AF (*n* = 18, 5.4%). Downstream bioinformatics analyses were performed to identify putative target genes, enriched gene ontology categories, and molecular pathways regulated by these candidate microRNAs.

**Results:**

We identified 77 extracellular RNAs (exRNAs) that were significantly associated with increased left ventricular (LV) mass and at least one additional echocardiographic intermediate phenotype. Among these, miR-17-5p and miR-574-3p were also significantly associated with a history of atrial fibrillation (AF), with odds ratios of 1.58 (95% CI: 1.10–2.26) and 2.16 (95% CI: 1.03–4.54), respectively. Predicted gene targets of these miRNAs were enriched in pathways implicated in atrial remodeling and arrhythmogenesis. Key overlapping canonical pathways included the Senescence Pathway, Idiopathic Pulmonary Fibrosis Signaling, ERK5 Signaling, RHO GTPase Cycle, and HGF Signaling.

**Conclusions:**

Circulating exRNAs, including miR-17-5p and miR-574-3p, are associated with cardiac remodeling and a history of AF in ACS survivors. These findings highlight their potential as biomarkers of atrial remodeling and implicate key molecular pathways involved in AF pathogenesis.

## Introduction

Atrial fibrillation (AF) is highly prevalent and is associated with significant morbidity, such as stroke and heart failure (1). Although, much is known about the sequelae about AF, the mechanisms by which AF arises and persists are not completely understood. Advancing our understanding of the maladaptive cellular and molecular responses that contribute to AF pathogenesis is critical for improving prevention and treatment strategies. Furthermore, the identification of robust biomarkers may enable risk stratification of individuals at elevated risk for the development of AF.

Small noncoding RNAs, particularly microRNAs (miRNAs), play pivotal roles in regulating cellular signaling pathways in both physiological and pathological contexts. MiRNAs are ∼22 nucleotide noncoding RNAs that modulate gene expression post-transcriptionally and have been shown to influence cardiac development, hypertrophy, fibrosis, and adverse remodeling in response to stressors (2, 3). Extracellular RNAs (ex-RNAs) are endogenous small noncoding RNAs that exist in the plasma with remarkable stability and may reflect cellular states and cellular communication (4). Ex-RNAs have emerged as promising biomarkers in cardiovascular disease, including AF. Although there are several reports implicating ex-RNAs in AF (5–7), few studies have analyzed miRNA profiles of patients with AF in the setting of acute coronary syndrome. Data illustrating the expression of plasma ex-RNAs in the acute clinical setting could provide relevant ex-RNA biomarkers and shed light on the molecular mechanisms underlying clinical AF.

Transthoracic echocardiography (TTE) is a widely utilized, noninvasive imaging modality that provides quantitative assessments of cardiac structure and function. It is an essential tool for the diagnosis, management, and prognostication of cardiovascular disease (8). Structural remodeling observed via echocardiography—such as increased left atrial size, reduced left ventricular ejection fraction (LVEF), and elevated left ventricular mass (LV mass)—has been consistently associated with prevalent and incident AF (9, 10). Furthermore, changes in echocardiographic phenotypes are associated with severity of the disease (11). The high utility of echocardiographic parameters in the evaluation AF severity is due to its ability to define structural processes underpinning pathological cardiac remodeling. Although echocardiographic phenotypes associated with AF are well known, the molecular basis for pathological cardiac remodeling is less understood.

To address this knowledge gap, we investigated the expression of circulating ex-RNAs in relation to echocardiographic indices of cardiac remodeling and clinical AF in a cohort of hospitalized ACS survivors. Using a two-step analysis strategy, we first identified ex-RNAs associated with echocardiographic phenotypes linked to pathological remodeling and subsequently examined their associations with prevalent AF. Our study leveraged data from the Transitions, Risks, and Actions in Coronary Events (TRACE-CORE) cohort, applying a mechanistic framework to nominate candidate ex-RNAs with potential roles in AF pathogenesis.

## Methods

### Study population

**Figure 1:**
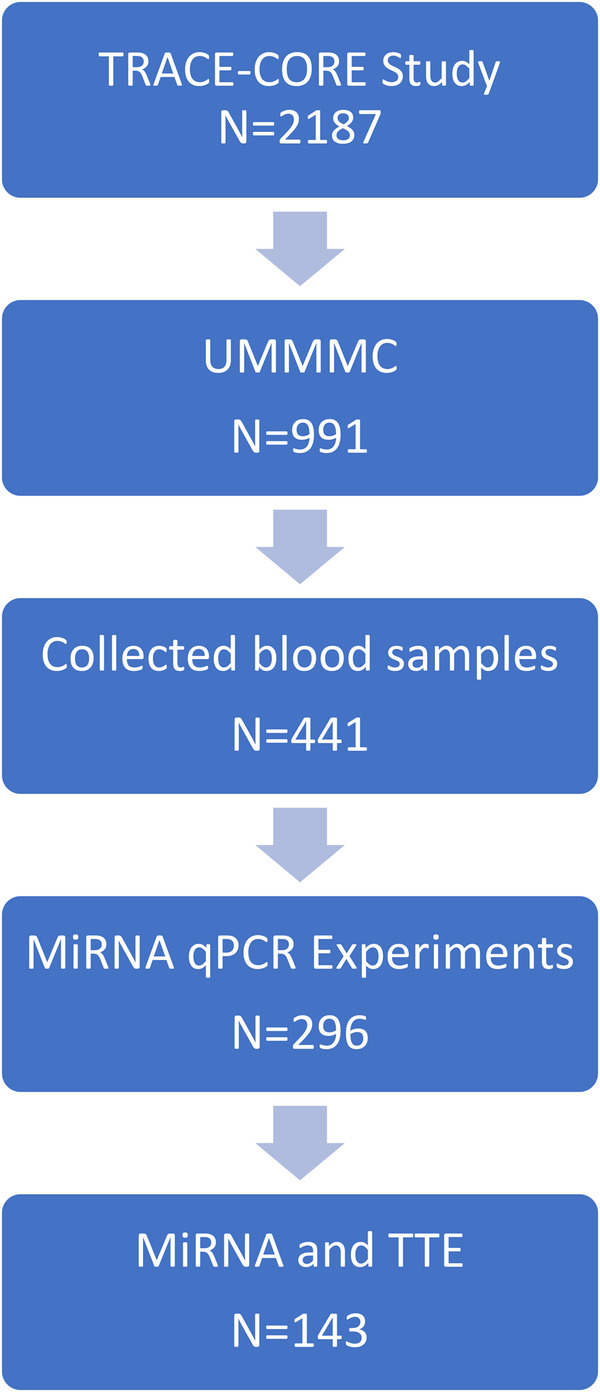
Sample selection for the analyses from the transitions, risks and action in coronary events (TRACE-CORE) study.

The design, participant recruitment strategy, interview protocols, and medical record abstraction methods employed in the Transitions, Risks, and Actions in Coronary Events (TRACE-CORE) study have been described in detail previously (12, 13). In brief, the TRACE-CORE study is a multicenter, prospective cohort study designed to examine recovery trajectories and long-term outcomes among patients hospitalized with acute coronary syndrome (ACS). TRACE-CORE utilized a six-site prospective cohort design to follow 2,187 patients discharged after an ACS hospitalization between April 2011 and May 2013 (Figure 1). In Massachusetts, participating sites included the two teaching hospitals (University and Memorial) that comprise UMass Memorial Medical Center (UMMMC), a large academic medical center, as well as St. Vincent Hospital, a major community hospital. These three hospitals provide care for the majority of ACS hospitalizations in central Massachusetts. In Georgia, sites included Northside and Piedmont Hospitals—community hospitals in Atlanta affiliated with Kaiser Permanente Georgia—and the Medical Center of Central Georgia, a major cardiac referral center located in Macon serving central and southern Georgia. At the sites in Central Massachusetts, 411 blood samples were collected, processed as described previously and plasma was stored in −80°C (4, 14). Of the plasma collected, 296 were of sufficient quality for RNA extraction and qPCR experiment. The institutional review boards at each participating recruitment site approved this study. All participants provided written informed consent.

### Ascertainment of AF

Trained study staff abstracted participants' baseline demographic, clinical, laboratory, and electrocardiographic data and in-hospital clinical complications from available hospital medical records. AF was identified through medical record abstraction during the index hospitalization, based on clinician documentation, electrocardiogram (ECG) findings, and telemetry reports. AF subtype (paroxysmal, persistent, or permanent) and episode duration were not recorded and thus were not captured in this analysis. Co-morbidities present at the time of hospital admission were identified from each participant's admission history and physical examination. Any patient with documentation of AF by a trained medical provider was considered to have prevalent AF.

### ex-RNA selection and profiling

As part of a transcriptomic profiling study, venous blood samples were collected during the index hospitalization from 296 participants enrolled in the TRACE-CORE cohort. Detailed protocols for blood processing, plasma storage, and RNA isolation have been described previously (14). Methods for the quantification of ex-RNAs, including miRNAs and small nucleolar RNAs (snoRNAs), have also been previously published (4). The panel of ex-RNAs was selected *a priori* based on prior data from the Framingham Heart Study (4). Plasma ex-RNA profiling was conducted at the High-Throughput Gene Expression & Biomarker Core Laboratory at the University of Massachusetts Medical School. Ex-RNA expression levels were reported as quantification cycle (Cq) values, with higher Cq values indicating lower transcript abundance. This approach resulted in the detection of 318 miRNAs. Additional methodological details are provided in the Supplementary Information (Supplementary Table S1).

### Echocardiographic measurements

Complete two-dimensional transthoracic echocardiograms were performed during the index hospitalization. Left ventricular ejection fraction (LVEF), volumetric parameters, and linear dimensions were assessed in accordance with the guidelines of the American Society of Echocardiography (ASE) (15). Quantified echocardiographic measures included left ventricular (LV) mass, LVEF, left ventricular end-diastolic (LVED) volume, left atrial (LA) volume, and left atrial volume index (LAVI) (Table 1). LV and LA volumes were derived using the Simpson's biplane method of disks from apical 2- and 4-chamber views. LV mass was calculated using the ASE-recommended formula: LV mass = 0.8 × [1.04 × (LVID + PWTd + SWTd)^3^—LVID^3^] + 0.6 g, where LVID is the LV internal diameter in diastole, PWTd is the posterior wall thickness in diastole, and SWTd is the septal wall thickness in diastole (15).

**Table 1:** Characteristics of TRACE-CORE participants included in the analytic sample.

Characteristics	No AF (*N* = 250)	AF (*N* = 46)	*P*-value
Age, mean SD	62.1 (11.5)	69.9 (10.0)	<0.001
Female	33.6	28.3	0.47
Race (Caucasian)	96.4	97.8	0.03
Height (inches)	67.9 (8.2)	75.4 (27.4)	0.0004
Weight (lbs)	188.1 (46.9)	183.6 (51.9)	0.59
Body Mass Index (kg/m^2^)	29.4 (5.5)	29.2 (5.8)	0.84
Social History			
Education			
High school	39.2	45.7	
Some college	28	23.9	0.7
College	32.8	30.4	
Married	66.00	67.4	0.85
Risk Factors			
Hyperlipidemia	67.6	71.7	0.58
Myocardial Infarction	28.8	34.8	0.42
Anginal Pectoris/CHD	26.00	37.00	0.14
Type 2 Diabetes Mellitus	28.8	28.3	0.94
Stroke/TIA	1.2	6.5	0.05
CHF	8.8	19.6	0.04
Hypertension	67.6	84.8	0.01
Seattle Angina Questionnaire			
Physical limitation	83.2 (22.0)	69.0 (27.6)	0.03
Angina stability	43.7 (40.3)	38.0 (23.5)	0.44
Angina frequency	75.4 (23.1)	67.7 (28.5)	0.19
Treatment satisfaction	93.9 (11.2)	93.0 (13.7)	0.77
Quality of life	64.8 (25.5)	54.8 (31.1)	0.12
Admission Medications			
Aspirin	46.0	63.0	0.03
Beta Blocker	38.4	69.6	<0.001
ACEI or ARB	36.8	56.5	0.01
Statin	54.8	80.4	0.0007
Plavix	13.6	15.2	0.77
Coumadin	2.00	30.4	<0.001
Physical Activity			
No physical acitivity	58.5	73.9	
<150 min/wk	15.5	15.2	0.05
>150 min/wk	26.00	10.9	
Acute Coronary Syndrome Category			
ST-elevation myocardial infarction	26.8	19.6	0.29
Physiological Factors			
Heart rate (beats per minute)	77.9 (19.4)	86.0 (31.2)	0.09
Systolic blood pressure (mmHg)	141.3 (25.1)	132.3 (23.2)	0.02
Diastolic blood pressure (mmHg)	79.5 (16.7)	75.1 (14.8)	0.07
Respiratory rate (breaths per minute)	18.1 (4.1)	19.3 (4.5)	0.11
Electrocardiogram			
QRS duration	96.7 (21.0)	100.2 (25.3)	0.39
PR interval	164.4 (26.9)	175.4 (47.3)	0.23
Lab Values			
Troponin peak	23.6 (35.9)	24.9 (34.7)	0.87
Total cholesterol	174.9 (46.5)	140.0 (39.5)	0.003
Brain natriuretic peptide	620.7 (853.2)	623.8 (428.7)	0.99
Creatinine	1.2 (0.51)	1.3 (0.55)	0.08
Hemoglobin	11.9 (2.15)	10.2 (2.27)	<0.001
Sodium	135.9 (3.3)	134.7 (3.6)	0.03
Echocardiographic Phenotype*			
LV Ejection Fraction	53.0 (12.7)	52.0 (12.2)	0.74
LA Volume	46.6 (17.7)	50.4 (30.3)	0.58
LAVI = LAVavg/BSA	23.7 (8.3)	24.3 (12.9)	0.82
LVIDd	4.8 (0.76)	4.7 (0.71)	0.58
LVIDs	3.39 (0.87)	3.41 (0.80)	0.94
GLS (−)	(−)13.4 (4.0)	(−)11.8 (4.1)	0.12
FSmmw	0.17 (0.06)	0.16 (0.03)	0.26
Fsen	0.32 (0.15)	0.29 (0.09)	0.16

* Echocardiographic phenotypes were characterized in subset of patients (*N* = 143) where TTE were available.

CHD, coronary heart disease; TIA, transient ischemic attack; ACEi, angiotensin-converting enzyme inhibitors; ARB, angiotensin II receptor blockers; LV, left ventricle; LA, left atrium; LAVI, left atrial volume index; LAVavg/BSA, average left atrial volume/body surface area.

### Statistical analyses

A two-step analysis model was used to leverage echocardiographic phenotypes to identify candidate ex-RNAs and then examining ex-RNAs identified and prevalent AF. In step 1, we examined the relations between ex-RNAs with one or more echocardiographic phenotypes (Table 2, Supplementary Table S2). In step 2, we examined the associations of ex-RNAs identified from step 1 with prevalent AF (Table 3). Given the modest sample size of our study, we did not adjust for age or other covariates. Of note, the number of participants in each step differed as we did not have echocardiographic data available for all participants with plasma ex-RNA data. There are 143 cases with both ex-RNA and echocardiographic data in our TRACE-CORE cohort (Figure 1). We used this group to determine the ex-RNAs significantly related to one or more echo parameters. Using this significant list of ex-RNAs, we queried for a relationship with prevalent AF on the full 296 cases with ex-RNA data.

**Table 2:** ex-RNAs associated with echocardiographic phenotypes.

ex-RNA	No AF (*N* = 250)				AF (*N* = 46)			
	*N*	Mean (1/Cq)	Median (1/Cq)	Std Dev	*N*	Mean (1/Cq)	Median (1/Cq)	Std Dev
hsa_miR_1_3p	78	0.0532116	0.0492086	0.021974	14	0.0486145	0.0485775	0.0021635
hsa_miR_10a_5p	74	0.0544765	0.0490171	0.026744	9	0.0479115	0.0471863	0.0020413
hsa_miR_10b_5p	105	0.0526464	0.0519433	0.005696	19	0.054176	0.0525778	0.008361
hsa_miR_1185_1_3p	10	0.076133	0.048098	0.077952	4	0.0811416	0.0646895	0.0489224
hsa_miR_1229_3p	87	0.0527826	0.0490296	0.023452	17	0.0496961	0.0494903	0.0030281
hsa_miR_1246	248	0.0697233	0.0689199	0.007744	46	0.0706521	0.0691848	0.0075221
hsa_miR_1247_5p	193	0.0530236	0.0512006	0.014657	30	0.0520521	0.0507146	0.0057972
hsa_miR_1271_5p	6	0.0711808	0.0462112	0.056029	3	0.1112173	0.0686812	0.0941748
hsa_miR_128_3p	102	0.0528408	0.0498578	0.020063	16	0.04953	0.0505303	0.0022436
hsa_miR_1307_3p	79	0.052133	0.0489367	0.01868	15	0.0487416	0.0481839	0.0027178
hsa_miR_139_5p	172	0.0509962	0.0504476	0.003436	31	0.0502585	0.0498191	0.0024643
hsa_miR_144_5p	91	0.0538011	0.0498353	0.034404	11	0.0504393	0.0503199	0.0020809
hsa_miR_152_3p	109	0.0546243	0.053788	0.015778	21	0.0531483	0.0531467	0.0048266
hsa_miR_17_3p	34	0.0581639	0.048011	0.048989	8	0.048671	0.0487127	0.0021114
hsa_miR_17_5p	174	0.0526555	0.0520309	0.00485	33	0.051199	0.0505799	0.0036006
hsa_miR_181c_3p	134	0.0496425	0.0493075	0.004581	19	0.0496737	0.0492549	0.0022402
hsa_miR_185_3p	12	0.0807253	0.0469793	0.078499	1	0.0467564	0.0467564	.
hsa_miR_186_5p	99	0.0496918	0.0492638	0.002817	23	0.0485152	0.0487082	0.0017551
hsa_miR_190a_3p	25	0.0559995	0.0481922	0.028327	5	0.0486872	0.0493391	0.0016668
hsa_miR_193b_3p	17	0.0474027	0.046771	0.002504	1	0.0494669	0.0494669	.
hsa_miR_194_5p	80	0.0514792	0.0489358	0.013029	13	0.0602008	0.0477006	0.0434223
hsa_miR_200b_3p	36	0.056391	0.0477348	0.028669	8	0.0535471	0.0482227	0.0116097
hsa_miR_200c_3p	28	0.0487998	0.0477602	0.003904	3	0.1384421	0.0479771	0.1585388
hsa_miR_210_3p	60	0.0482735	0.0479058	0.002153	9	0.0485037	0.0484956	0.0019539
hsa_miR_2110	66	0.049364	0.0483657	0.007079	6	0.0477354	0.0471473	0.0024344
hsa_miR_215_5p	48	0.0611305	0.0487926	0.067106	6	0.0524814	0.0511999	0.0057445
hsa_miR_22_3p	194	0.052095	0.0517429	0.003923	35	0.0524587	0.0512132	0.0065555
hsa_miR_22_5p	58	0.0507662	0.0482315	0.007676	11	0.0537059	0.0484255	0.0141602
hsa_miR_224_5p	88	0.0490206	0.0483175	0.002941	10	0.0501532	0.0494765	0.0031774
hsa_miR_296_5p	119	0.0607483	0.0636846	0.01262	23	0.0623518	0.0669331	0.0114245
hsa_miR_29a_3p	186	0.0542824	0.0527513	0.009596	32	0.0643896	0.0526593	0.061423
hsa_miR_29b_3p	100	0.0497822	0.0492949	0.002587	11	0.0619879	0.0482569	0.0456338
hsa_miR_29c_3p	145	0.051691	0.0511776	0.003584	27	0.050784	0.0504793	0.0028985
hsa_miR_29c_5p	247	0.0586745	0.0605409	0.005869	46	0.0585694	0.0597602	0.0055613
hsa_miR_301b_3p	34	0.0570262	0.0489981	0.021454	8	0.0505417	0.0460105	0.0115485
hsa_miR_30b_5p	104	0.0524204	0.049863	0.013113	19	0.0518143	0.0490891	0.0078867
hsa_miR_30c_5p	158	0.0536866	0.050752	0.028003	31	0.0506949	0.049967	0.0029418
hsa_miR_320d	34	0.0474423	0.0467811	0.001899	7	0.0475848	0.0474307	0.0014531
hsa_miR_324_3p	195	0.0518799	0.052044	0.003289	39	0.0509503	0.0515589	0.0032071
hsa_miR_331_3p	76	0.0581103	0.0590573	0.00509	15	0.057876	0.0590844	0.0039275
hsa_miR_337_3p	49	0.0544614	0.049184	0.022267	6	0.0501628	0.0496202	0.0020578
hsa_miR_342_5p	26	0.0540327	0.0477244	0.031145	3	0.0458949	0.0457851	0.0003425
hsa_miR_34a_3p	42	0.0481224	0.0475388	0.003913	7	0.0478629	0.04801	0.0017482
hsa_miR_3615	159	0.0502273	0.0493263	0.009675	27	0.048445	0.0482488	0.0018838
hsa_miR_378a_3p	142	0.0518858	0.0506277	0.013493	25	0.0503217	0.0495978	0.0044944
hsa_miR_381_3p	6	0.055495	0.0521887	0.012588	2	0.0531945	0.0531945	0.0033558
hsa_miR_425_5p	65	0.0518003	0.0493772	0.009402	9	0.0484625	0.0477814	0.0020986
hsa_miR_4446_3p	238	0.0574881	0.0596986	0.006702	44	0.0572826	0.0593641	0.0066937
hsa_miR_450b_5p	37	0.0590072	0.0480829	0.035178	4	0.0836709	0.0504592	0.0687618
hsa_miR_454_3p	48	0.0607224	0.0486327	0.035194	6	0.0496523	0.0488364	0.003395
hsa_miR_4770	28	0.0724879	0.0492878	0.04995	10	0.0641504	0.0490861	0.0419758
hsa_miR_483_3p	33	0.0612955	0.0470936	0.044739	6	0.0487458	0.0488686	0.00068702
hsa_miR_497_5p	62	0.0507918	0.048863	0.008547	7	0.0502849	0.0504037	0.002183
hsa_miR_502_3p	35	0.0505644	0.0478932	0.013581	3	0.0465949	0.0467552	0.00060646
hsa_miR_532_3p	192	0.052351	0.0502476	0.005461	33	0.0539177	0.0530617	0.0070026
hsa_miR_532_5p	38	0.0523519	0.0475867	0.017305	3	0.1580497	0.1890047	0.0857976
hsa_miR_545_5p	9	0.0822204	0.0489364	0.07149	3	0.093437	0.1052632	0.0404737
hsa_miR_548d_3p	25	0.0477521	0.0475845	0.00138	9	0.0468183	0.046468	0.0010111
hsa_miR_548e_3p	16	0.10365	0.0483893	0.118834	3	0.0794202	0.0669776	0.041042
hsa_miR_550a_3p	6	0.0882057	0.0489723	0.096385	1	0.0460695	0.0460695	.
hsa_miR_574_3p	89	0.0519335	0.049021	0.017758	19	0.0488315	0.0484904	0.0026017
hsa_miR_582_3p	12	0.0733303	0.0561478	0.04628	1	0.0614546	0.0614546	.
hsa_miR_584_5p	153	0.0535996	0.0504307	0.028997	28	0.0505161	0.0509117	0.0026293
hsa_miR_590_3p	19	0.0490983	0.0483512	0.002403	1	0.0726819	0.0726819	.
hsa_miR_590_5p	25	0.0727524	0.0493378	0.062432	3	0.1036606	0.1119633	0.0543337
hsa_miR_642a_5p	15	0.1042246	0.0928847	0.091516	2	0.0912716	0.0912716	0.0482923
hsa_miR_654_3p	31	0.0853935	0.0548196	0.06543	4	0.0599797	0.0572262	0.0140572
hsa_miR_654_5p	24	0.0481984	0.0477808	0.002624	2	0.0491084	0.0491084	0.00107
hsa_miR_659_3p	45	0.0474394	0.0475009	0.001371	7	0.0472299	0.0474096	0.00044278
hsa_miR_660_5p	91	0.0524652	0.0491282	0.020054	16	0.0497289	0.0488174	0.0025629
hsa_miR_664a_5p	34	0.0530387	0.0468396	0.031089	4	0.0481101	0.0479805	0.0019548
hsa_miR_6803_3p	38	0.0492133	0.047891	0.007958	8	0.0808498	0.0494892	0.0907363
hsa_miR_7977	50	0.048617	0.0483476	0.001892	5	0.0480875	0.048202	0.0017095
hsa_miR_877_3p	66	0.0532129	0.0504439	0.015183	15	0.050662	0.050671	0.0025874
hsa_miR_885_5p	65	0.0546778	0.0486122	0.022415	10	0.0483796	0.0479623	0.0018983
hsa_miR_9_3p	74	0.0513739	0.0497939	0.006949	12	0.0520303	0.0518041	0.0039796
hsa_miR_99a_5p	192	0.0549907	0.0558302	0.004637	33	0.0552821	0.0562075	0.0046839

**Table 3:** miRNAs significantly related to history of AF.

miRNA	*n*	mean	std	Estimate	StdErr	ProbChiSq	OddsRatioEst	LowerCL	UpperCL	Raw *P*-value	FDR *P*-value
hsa_miR_106b_5p	171	19.6432	1.426	0.5328	0.2517	0.0343	1.704	1.04	2.79	0.0343	0.0395
hsa_miR_125b_5p	126	20.0642	1.00202	1.0726	0.4102	0.0089	2.923	1.308	6.53	0.0089	0.0343
hsa_miR_142_3p	81	19.5754	3.04565	1.6648	0.8318	0.0454	5.285	1.035	26.982	0.0454	0.0454
hsa_miR_150_5p	269	16.2008	2.00911	0.2283	0.0993	0.0214	1.256	1.034	1.526	0.0214	0.0343
hsa_miR_15b_3p	175	19.4343	1.57645	0.6897	0.2322	0.003	1.993	1.264	3.142	0.003	0.0343
**hsa_miR_17_5p**	**207**	**19**.**2059**	**1**.**49941**	**0**.**4549**	**0**.**1828**	**0**.**0128**	**1**.**576**	**1**.**101**	**2**.**255**	**0**.**0128**	**0**.**0343**
hsa_miR_20b_5p	199	18.5883	2.30551	0.482	0.2119	0.0229	1.619	1.069	2.453	0.0229	0.0343
hsa_miR_23b_3p	186	19.5649	1.5271	0.5582	0.2394	0.0197	1.748	1.093	2.794	0.0197	0.0343
hsa_miR_3613_3p	243	18.4669	1.6719	0.3741	0.144	0.0094	1.454	1.096	1.928	0.0094	0.0343
hsa_miR_362_3p	27	18.5678	4.59545	−0.3338	0.1403	0.0174	0.716	0.544	0.943	0.0174	0.0343
hsa_miR_433_3p	220	19.3035	2.34432	−0.1656	0.0752	0.0276	0.847	0.731	0.982	0.0276	0.0376
hsa_miR_495_3p	115	19.7469	1.49644	0.9642	0.3822	0.0116	2.623	1.24	5.547	0.0116	0.0343
**hsa_miR_574_3p**	**108**	**20**.**0059**	**1**.**99826**	**0**.**773**	**0**.**3771**	**0**.**0404**	**2**.**166**	**1**.**034**	**4**.**536**	**0**.**0404**	**0**.**0433**
hsa_miR_6511b_3p	138	20.3203	1.113	0.7134	0.337	0.0343	2.041	1.054	3.951	0.0343	0.0395
hsa_miR_942_5p	84	20.4355	1.65312	−0.5972	0.2437	0.0143	0.55	0.341	0.887	0.0143	0.0343

Bolded miRNA are also associated with echophenotypes.

For step 1 of our analyses, we used ordinary least-squares linear regression to quantify associations between ex-RNA levels and one or more echocardiographic phenotypes in all participants. To account for multiple testing, we employed Bonferroni correction to establish a more restrictive threshold for defining statistical significance. We established a 5% false-discovery rate (via the Benjamini-Hochberg false discovery rate approach) to screen associations between ex-RNAs and one or more echocardiographic phenotypes. The α for achieving significance was set at 0.05/340 = 0.000147 *a priori*. Note that C_q_ represents a log measure of concentration, with exponentiation factor 2. In step 2 of the analysis, we examined the associations of miRNAs identified from step 1, with prevalent AF using a logistic regression model. Here, the continuous 1/C_q_ values, which corresponded to plasma miRNA levels, were compared with prevalent AF (Table 3).

Differentially expressed miRNAs were analyzed using miRDB, an online database that captures miRNA and gene target interactions (16, 17). The network and functional analyses were generated through the use Qiagen's Ingenuity Pathway Analysis (IPA) version 24.0.2 (18). All statistics were performed with SAS software version 9.3 (SAS Institute) with a 2-tailed *P* value < 0.05 as significant.

## Results

### Patient characteristics

The baseline demographic, clinical, and echocardiographic characteristics of the 296 study participants are outlined in Table 1. Study participants were middle aged to older adults (mean age of 62 ± 11 and 70 ± 10 for the no AF [control group] vs. the AF group, respectively). There was a male predominance; women represented 33% and 28% of control and AF groups, respectively. The patients with AF had a significant higher history of transient ischemic attacks, strokes, congestive heart failure and hypertension (Table 1). There are nonsignificant trends of higher LA volume (46.6 vs. 50.4 ml) and lower global longitudinal strains (−13.4% vs. 11.1%) in patients with AF as compared to those without (Table 1). However, we did not find any significant trends in in echo parameters associated with and without AF.

### Association of ex-RNAs with echocardiographic phenotypes and AF

A total of 318 ex-RNAs were quantified in the plasma of TRACE-CORE participants included in our investigation. There were 77 ex-RNAs that associated with one or more echocardiographic parameters, independent of other clinical variables (Table 2). Five miRNAs were associated with three or more echocardiographic traits, miR-10a-5p, miR-10b-5p, miR-190a-3p, miR-425-5p, and miR-574-3p (Supplementary Table S2). Of the 77 ex-RNA that were significantly associated echo-phenotypes, miR-17-5p and miR-574-3p were also significantly associated with a history of AF, with odds ratios of 1.58 (95% CI: 1.10–2.26) and 2.16 (95% CI: 1.03–4.54), respectively (Table 3). We found fifteen ex-RNAs that associated with prevalent AF via unadjusted logistic regression modeling (Table 3).

### Gene targets of ex-RNAs associated with prevalent AF

**Figure 2:**
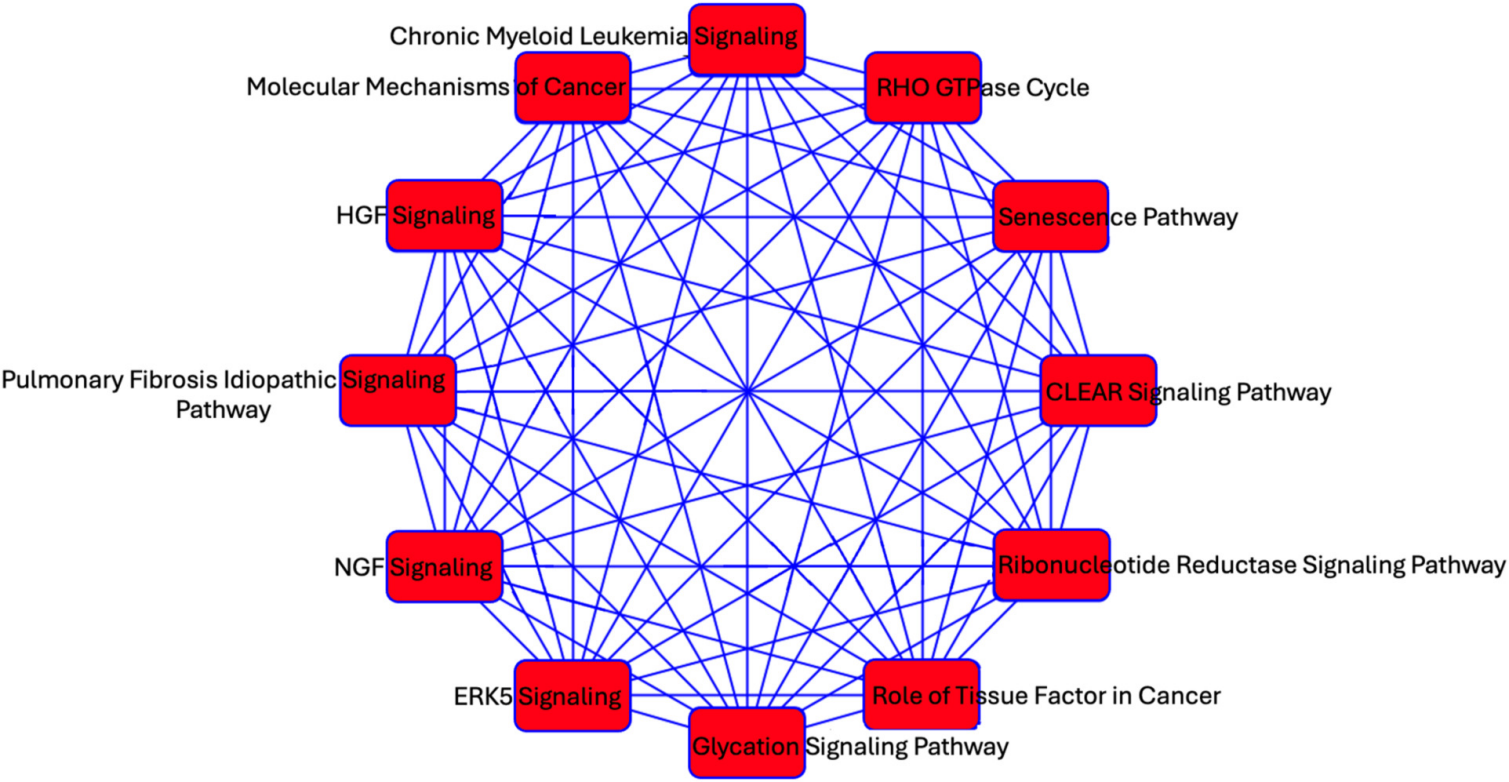
A network analysis of predicted targets of miR-17-5p and miR-574-3p as performed by IPA. Each node represents a unique pathway, and edges denote shared gene components between pathways. The pathways include those implicated in fibrosis (*Idiopathic Pulmonary Fibrosis Signaling Pathway*), cellular stress responses (*ERK5 Signaling,* RHO GTPase signaling, *Senescence Pathway*), cancer (e.g., *Molecular Mechanisms of Cancer*, *Chronic Myeloid Leukemia Signaling*), and metabolic regulation (*Glycation Signaling Pathway*).

We investigated predicted targets of the two miRNAs associated with echocardiographic phenotypes and prevalent AF using the database miRDB. From this, 1,355 genes were predicted as targets for at least one miRNA. As miRNA are known to act in concert, we used the combined targets of miR-17-5p and miR-574-3p to perform further analysis in IPA (2). Overlapping canonical pathways were mapped to allow for visualization of the shared biological pathways through the common genes (Figure 2). The resulting network revealed significant enrichment of interconnected canonical pathways, including the Senescence Pathway, Idiopathic Pulmonary Fibrosis Signaling Pathway, RHO GTPase Cycle, ERK5 Signaling, HGF Signaling, NGF Signaling, CLEAR Signaling Pathway, Glycation Signaling Pathway, Chronic Myeloid Leukemia Signaling, Molecular Mechanisms of Cancer, and Role of Tissue Factor in Cancer.

## Discussion

In our investigation of ex-RNA profiles of 296 hospitalized ACS survivors in the TRACE-CORE Cohort, we identified 77 plasma ex-RNAs associated with one or more echocardiographic traits. Furthermore, two of these ex-RNAs, miR-17-5p and miR-574-3p, were associated with prevalent AF. While the association of miRNA and AF has been explored previously, our study uniquely examined the association between ex-RNA and AF in the acute clinical setting. We identified miR-17-5p and miR-574-3p as regulators in cardiac remodeling and AF in patients hospitalized for ACS.

### Echocardiographic phenotypes and cardiac remodeling in AF

AF is intimately linked with structural and functional remodeling of the heart, particularly involving the left atrium and ventricle. Among the echocardiographic parameters that reflect these changes, the left atrial volume index (LAVI) and global longitudinal strain (GLS) are particularly sensitive indicators of remodeling. In the presented data, there is a slight but consistent trend toward increased LAVI in the AF group, suggesting chronic atrial pressure or volume overload. While this subtle enlargement may not reach statistical significance, it likely reflects early or sustained atrial remodeling associated with AF pathophysiology. Elevated LAVI has been associated with increased atrial stiffness and fibrosis, both of which are known contributors to the maintenance and recurrence of AF (19, 20).

Simultaneously, a downward trend in GLS values in the AF group indicates early LV systolic dysfunction despite preserved ejection fraction. GLS, which captures longitudinal myocardial deformation, is a more sensitive marker of subclinical ventricular dysfunction than traditional metrics like LVEF. Reduced GLS in AF patients, even in the absence of overt systolic impairment, underscores the subtle mechanical changes that accompany electrical abnormalities in this arrhythmia (21). These findings suggest that the combination of mild increases in LAVI and early reductions in GLS may serve as echocardiographic markers of the atrial and ventricular remodeling continuum seen in AF (11).

### Association of ex-RNAs, cardiac remodeling and AF

Several canonical pathways enriched among the predicted targets of miR-17-5p and miR-574-3p converge on well-established mechanisms of atrial remodeling. The Senescence Pathway and Idiopathic Pulmonary Fibrosis (IPF) Signaling Pathway are central to fibrotic transformation and extracellular matrix accumulation, processes that underlie the adverse structural substrate. Senescent fibroblasts and cardiomyocytes can adopt a pro-inflammatory secretory phenotype, amplifying fibrotic signaling and promoting atrial electrical inhomogeneity (22, 23). Similarly, the ERK5 and RHO GTPase signaling pathways are activated in response to mechanical stress and oxidative injury—conditions prevalent in the atria of patients with elevated atrial pressure or volume overload—and are implicated in cytoskeletal reorganization, endothelial dysfunction, and atrial dilation (24, 25). Furthermore, dysregulation of the Hepatocyte Growth Factor (HGF) Signaling Pathway, while generally cardioprotective, may lead to increased vulnerability to AF (26).

In addition, NGF signaling plays a key role in autonomic remodeling, a known contributor to AF initiation and maintenance. Elevated NGF levels in atrial tissue have been associated with sympathetic hyperinnervation and parasympathetic imbalance, both of which shorten atrial refractoriness and promote arrhythmogenicity (27). Interestingly, the enrichment of cancer-associated pathways such as Chronic Myeloid Leukemia Signaling, Molecular Mechanisms of Cancer, and Role of Tissue Factor in Cancer may reflect shared transcriptional programs between fibrotic and proliferative remodeling in AF and oncogenic processes (28, 29). Together, the convergence of these pathways suggests a multifactorial molecular basis for AF, in which structural, autonomic innervation, and inflammatory mechanisms intersect.

Emerging evidence suggests that miR-17-5p and miR-574-3p play a role in the pathogenesis of AF. miR-17-5p is part of the miR-17-92 cluster, which has been shown to regulate genes involved in sinoatrial node development and pacemaker activity (30). Specifically, miR-17-92 represses genes such as Shox2 and Tbx3, which are crucial for sinoatrial node formation. Deficiency of miR-17-92 leads to increased susceptibility to pacing-induced AF and sinoatrial node dysfunction—both recognized as risk factors for AF in humans (30). Similarly, miR-574-3p has been shown to be elevated in the left atrial appendages of patients with persistent AF secondary to mitral stenosis, compared to those in normal sinus rhythm, suggesting its involvement in adverse structural remodeling (31).

### Strength and limitations

This study has several strengths. We analyzed associations between circulating ex-RNAs and both echocardiographic phenotypes and AF in a well-characterized cohort of patients hospitalized with ACS as part of the TRACE-CORE study. This cohort uniquely enabled the profiling of plasma ex-RNA expression in the acute clinical setting. While it is conceivable that the observed ex-RNA signatures may be influenced by the acute ischemic event rather than AF, our prior work did not identify significant ex-RNA differences attributable to acute myocardial infarction (32). Given that we employed a similar analytic method in the current study, the differential ex-RNA expression is more plausibly attributed to AF status rather than ACS event.

Several limitations should be acknowledged. The relatively small sample size and limited racial and geographic diversity may constrain the generalizability of our findings. Moreover, while we identified several microRNAs associated with echocardiographic parameters and AF, the cellular origin and biological mechanisms underlying their release into the circulation remain undefined. In addition, AF subtypes were not systematically recorded and could not be assessed in this analysis. Due to the modest sample size of our study, we did not adjust for age or other covariates, which limits our ability to evaluate independent associations between ex-RNA expression and AF. Future studies incorporating mechanistic investigations, including cellular and molecular approaches, as well as multivariable models to account for potential confounders such as age are needed to elucidate the functional roles of these miRNAs in AF pathogenesis.

## Conclusion

In this study of hospitalized ACS survivors, we identified circulating ex-RNAs, including miR-17-5p and miR-574-3p, that are associated with echocardiographic markers of cardiac remodeling and with prevalent atrial fibrillation. These miRNAs target gene networks involved in fibrosis, senescence, cytoskeletal remodeling, and autonomic signaling—pathways central to the structural and electrical substrate of AF. Our findings suggest that plasma ex-RNAs may serve as informative biomarkers of atrial remodeling and AF risk, particularly in the acute clinical setting. By linking molecular signatures with imaging phenotypes, this study provides a foundation for further investigation into the mechanistic roles of ex-RNAs in AF pathogenesis and their potential utility in risk stratification and therapeutic targeting.

## Data Availability

The raw data supporting the conclusions of this article will be made available by the authors, without undue reservation.
